# Editorial: Senescence and reprogramming: hallmarks in aging and disease

**DOI:** 10.3389/fcell.2025.1622682

**Published:** 2025-05-16

**Authors:** F. Javier González-Rico, Jiyue Zhu, Guangyong Peng

**Affiliations:** ^1^ Departamento de Bioquímica y Biología Molecular y Genética, Facultad de Ciencias, Universidad de Extremadura, Badajoz, Spain; ^2^ Department of Pharmaceutical Sciences, Washington State University, Spokane, WA, United States; ^3^ Department of Otolaryngology-Head and Neck Surgery, Washington University School of Medicine, Saint Louis, MO, United States

**Keywords:** reprogramming, aging, senescence, OSKM, cancer, transdifferentiation, progeroid syndromes

Aging is a biological process marked by the gradual decline of molecular, cellular and tissue functionality. This progressive degeneration is intrisically linked to advancing age and contributing to the development of age-related diseases, such as cancer, cardiovascular diseases, neurodegenerative conditions, osteoporosis, kidney and lung failure, among others.

The twelve hallmarks driving the aging process are genomic instability, telomere attrition, epigenetic alterations, loss of proteostasis, disabled macroautophagy, deregulated nutrient-sensing, mitochondrial dysfunction, cellular senescence, stem cell exhaustion, altered intercellular communication, chronic inflammation, and dysbiosis (López-Otín et al., 2023). These hallmarks share three defining characteristics: they manifest during physiological aging, their experimental exacerbation accelerates aging, and their mitigation through intervention slows aging, thereby extending healthy lifespan (Tartiere et al., 2024).

The aging-associated characteristics are closely linked to cellular senescence, a state defined by cell cycle arrest, spatial chromatin reorganization, and alterations in gene expression patterns. These changes also lead to the secretion of proinflammatory cytokines, proteases and growing factors, collectively known as Senescence-Associated Secretory Phenotype (SASP) (Campisi and d’Adda di Fagagna, 2007). A key driver of replicative cellular senesence is the progressive shortening of telomeres that occurs with aging. Additionally, cells can enter senescence in response to various stressors, including sustained mitogenic signaling, oncogene activation, DNA damage from irradiation, oxidative and genotoxic stress, epigenetic alterations, chromatin disorganization, perturbed proteostasis, mitochondrial dysfunction, inflammation signals, tissue damage, chemotherapeutic agents, and nutrient deprivation (Kumari and Jat, 2021).

Both cellular senescence and organismal aging are closely connected to epigenetic states. During development, epigenetic modifications, including DNA methylation, histone modifications, and chromatin remodeling, collectively determine cellular differentiation status and cell fates. These differentiation states can also be reversed through the transcriptional and/or epigentic reprogramming of the cell. In cell reprogramming, the cell identity and plasticity can be altered by the overexpression of transcription factors such as Oct4, Sox2, Klf4 and c-Myc (OSKM) (Takahashi and Yamanaka, 2006). This induction creates a dedifferentiation state that enhances tissue regeneration and has been succesfully applied in various pathological contexts, including diabetes, muscle injuries, retinal degeneration, and myocardial infarction (Ocampo et al., 2016; Lu et al., 2020; Chen et al., 2021). Those studies reveal the potential of cell reprogramming tools for controlling cell fate and function for disease therapies.

Within this broad context, we present an article Research Topic for Frontiers under the Research Topic “Senescence and Reprogramming: Hallmarks in Aging and Disease”, offering expert and up to date perspective on aging, senescence and reprogramming, encompassing both original research and review articles.

In this Research Topic, Cai et al. present an original research investigating the application of electromagnetic fields (EMF) to rejuvenate aged bone marrow mesenchymal stem cells (BMSCs). They established a robust experimental model in the field of tissue engineering, demonstrating potential for stem cell therapy. Their detailed methodological description, complemented by rigorous controls, provides a valuable tool for research groups studying stem cell aging.

Chen et al. elucidate the role of Vitamin D in regulating granulosa cell aging and homone balance. Using the Vitamin D receptor (VDR) deficiency mice, they showed the importance of VDR in follicle maturation and hormone secretion, identifying its deficiency as a driver of premature ovarian insufficiency. Their study further demonstrates that *Vdr* deficiency accelerates granulosa cell aging through the antioxidant and anti-aging effect of 7-dehydrocholesterol (7-DHC). Employing both human and murine models, they apply a comprehensive methodological approach, including genome editing for knockout generation, chromatin immunoprecipitation, and intracelular ROS analysis via flow cytometry.

Keshri et al. contribute a review article discussing emerging strategies in direct reprogramming, addressing challenges related to efficacy, cellular maturity, and targeting specificity. Their work covers multiple aspects of transdifferentiation, including signalling pathways, epigenetic modifications, and metabolic regulation. They also discussed next-generation tools in reprogramming, such as minibinders, EpiBinders, CRISPR-Cas9, and chromatin modulation by pioneer transcription factors.

In a complementary perspective, Moledo-Nodar et al. examine progeroid syndomes, emphasizing the therapeutic promise of partial reprogramming strategies that erase age-associated cellular characteristics while preserving identity. Given the phenotypic overlap between progeroid syndromes and physiological aging, their discussion underscores both the potential and the challenges inherent in applying reprogramming-based therapies to these rare genetic disorders.

Finally, Sinenko et al. reviewed the molecular mechanisms governed by and influencing metabolic pathways during the reprogramming process. Their comprehensive analysis discussed the stages of cell reprogramming toward pluripotency in both human and mouse induced pluripotent stem cells (iPSCs), with a particular focus on metabolic profiles and the transcription factors and pathways involved.

We hope that the articles featured in this Research Topic will serve as valuable resources for researchers in the fields of aging, senescence, and cell reprogramming, whether they are well-established experts or newcomers eager to explore this exciting arena.
